# Thyroid carcinoma after Chernobyl latent period, morphology and aggressiveness

**DOI:** 10.1038/sj.bjc.6601860

**Published:** 2004-05-11

**Authors:** E D Williams, A Abrosimov, T Bogdanova, E P Demidchik, M Ito, V LiVolsi, E Lushnikov, J Rosai, Yu Sidorov, M D Tronko, A F Tsyb, S L Vowler, G A Thomas

**Affiliations:** 1 Thyroid Carcinogenesis Research Unit, Strangeways Research Laboratory, Worts Causeway, Cambridge CB1 8RN, UK; 2 MRRC RAMS Obninsk, 249020 Kaluga Obl, Russian Federation; 3 Institute of Endocrinology and Metabolism, Vyshgorodskaya Str. 69, Kiev 254114, Ukraine; 4 Research Institute for Radiation Medicine and Clinical Endocrinology, Masherov Avenue 23, Minsk 220600, Belarus, Ukraine; 5 Department of Pathology, National Nagasaki Medical Centre, 2-1001-1-Kubara, Omura, Nagasaki 856-8562, Japan; 6 Department of Pathology and Laboratory Medicine, University of Pennsylvania Medical Centre, 3400 Spruce Street, Philadelphia 19104, USA; 7 Department of Pathology, Istituto Nazionale Tumori, Via Venezian 1, Milano 20133, Italy; 8 Centre for Applied Medical Statistics, Institute of Public Health, University of Cambridge, Forvie Site, Robinson Way, Cambridge CB2 2SR, UK; 9 South West Wales Cancer Institute, Singleton Hospital, Swansea SA2 8QA, UK

**Keywords:** thyroid carcinoma, tumour latency, Chernobyl, RET

## Abstract

The large numbers of papillary thyroid carcinomas that have occurred in those exposed to high levels of short-lived isotopes in fallout after Chernobyl provide a unique opportunity to correlate latency and tumour biology. We show that short latency is associated with tumours with a phenotype that is significantly less structurally differentiated, shows significantly less peritumour fibrosis, and significantly more invasive spread when compared to tumours with a longer latent period. In contrast, the type of differentiation (papillary or follicular architecture) is associated with age at exposure. These findings suggest that the initial mutation at the time of exposure played a major role in tumour latency and aggressiveness. We and others have shown that RET-PTC3 rearrangements are associated with the solid morphology seen in these short latency tumours, while classical papillary carcinomas more often show RET-PTC1 rearrangements. Studies in transgenic mice show similar findings, and *in vitro* studies show that RET-PTC3 induces more rapid growth than RET-PTC1. We therefore suggest that the solid morphology, high frequency of RET-PTC3 rearrangements and aggressive behaviour noted in early investigations of post-Chernobyl tumours were characteristic of short latency rather than the nature of the mutagen, and that successive overlapping waves of papillary carcinoma with differing latency, differing patterns of mutations and differing clinical behaviour are occurring in those exposed to Chernobyl fallout.

The accident at the Chernobyl nuclear power plant in April 1986 led to the exposure of millions of people in the surrounding areas to high levels of fallout (Shigematsu, 1991). An increase in the incidence of thyroid carcinoma in children was first noticed 4 years after the accident (Baverstock *et al*, 1992; Kazakov *et al*, 1992), and those exposed as children continue to show an increased incidence of thyroid carcinoma (UNSCEAR, 2000). In Belarus, Northern Ukraine and parts of the Russian Federation, about 2000 cases of thyroid carcinoma can be attributed to exposure to fallout; nearly all are papillary carcinomas, and together they form the largest number of cases of cancer of a single type due to a known cause on a known date that have ever occurred (Williams, 2002). The cases vary in their age at exposure and latency; although over 95% of the cancers are classified as papillary carcinomas, they vary also in their morphology, ranging from a solid immature phenotype, lacking typical architectural differentiation and with limited thyroglobulin content, to well-differentiated tumours dominated by either papillary or follicular architecture (Furmanchuk *et al*, 1992; Nikiforov and Gnepp, 1994; Bogdanova *et al*, 1995; Williams, 1996; Tronko *et al*, 1999). Many tumours contain a mixture of different patterns. Owing to the 8-day half-life of ^131^I, the main isotope involved, the period of radiation exposure was relatively brief, so that these Chernobyl-related carcinomas provide an unparalleled opportunity to link the morphological features, molecular biological findings and clinical characteristics to the age at exposure and latent period.

The major oncogene known to be involved in the genesis of papillary carcinoma of the thyroid is RET, although more recently BRAF has also been identified in a significant proportion of adult cases (Kimura *et al*, 2003; Soares *et al*, 2003). RET is activated in papillary carcinoma by rearrangement of its tyrosine kinase domain (Santoro *et al*, 1992). A variety of rearrangements occur, but RET-PTC 1 and RET-PTC 3, involving rearrangement to the H4 and ELE1 genes, respectively, are the most common. We and others have already shown that tumours with a major solid component are more often positive for a RET-PTC 3 arrangement, while tumours that are dominated by a well-differentiated papillary component more often show a RET-PTC 1 rearrangement (Nikiforov *et al*, 1997; Thomas *et al*, 1999; Rabes *et al*, 2000). In the present study, we set out to investigate possible associations between the morphological features of tumour architecture and aggressiveness, with age at exposure, age at operation and latent period.

## MATERIALS AND METHODS

**Figure 1:**
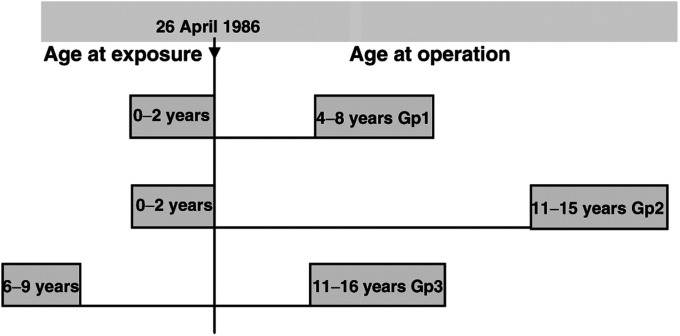
Graphic representation of ages at exposure and at operation for the three groups and their relationship to the Chernobyl accident.

**Figure 2:**
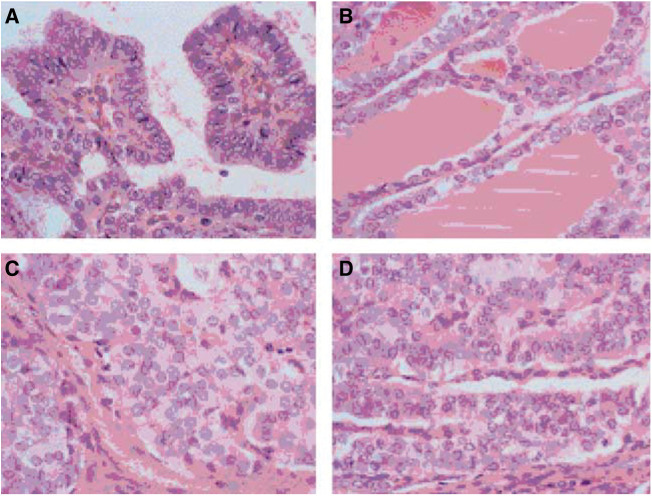
Typical examples of the types of tumour morphology analysed: (**A**) papillary, (**B**) follicular, (**C**) solid and (**D**) trabecular.

Tumours for study were chosen randomly within three groups defined by age at exposure and age at operation. All were papillary carcinomas and were from exposed areas in Belarus, Ukraine and the Russian Federation. Group one included 29 cases aged under 2 years at exposure and 4–8 years at operation, group two 35 cases aged under 2 years at exposure and 11–15 years at operation, and group three 26 cases aged 6–9 years at exposure and 11–16 years at operation. (Figure 1

). On review, four cases were excluded because the available material was only from metastases. To provide a quantitative assessment of the morphological features, tumour sections from each case were studied by a group of eight pathologists each of whom scored a series of features using an agreed protocol. Each of the architectural patterns present in the tumour (papillary, follicular, solid or trabecular) (Figure 2

) was scored for each tumour, either as absent, minor (<10%), moderate (11–50%) or dominant (51–100%). The degree of intrathyroid invasion was scored on a three-point scale (absent, moderate, marked), and the presence or absence of extrathyroid invasion was recorded. Tumour-associated fibrosis was divided into intratumour fibrosis, and peritumour (capsular) fibrosis; each was scored on a three-point scale as before.

The data for the tumour architecture was analysed using the mid-range score for each component, corrected to bring each observers total to 100%. The mean score for all participants was then determined for each tumour. The mean score for the tumour-associated categories and for intrathyroid invasion was calculated using a mark of 0, 1 and 2 for absent, moderate, marked, respectively. For each of these variables, groups were compared using the Kruskal–Wallis test. Where this was significant, the Mann–Whitney *U* was used in *post hoc* tests. For extrathyroid invasion, the modal class for each tumour was determined, that is the category listed by the largest number of observers, and groups compared using the χ^2^ test. The results are presented in the text as means with 95% confidence limits and in the figures as box and whisker plots showing medians and interquartile ranges.

The aim of the methodology adopted was to achieve a quantitative assessment of the type and extent of the morphological features present. This required a combination of a qualitative judgement in distinguishing the components and a quantitative assessment of their extent. The purpose was to arrive at a consensus estimate rather than compare the reproducibility of individual assessors results, nevertheless a kappa analysis was carried out. With the exception of the trabecular component, which was present in very small amounts, all kappa values lay between 0.40 and 0.56, a moderate level of agreement.

## RESULTS

### Characteristics of the three groups

Group 1 consisted of 28 cases (16F, 12M) exposed in infancy with a 6-year latent period before operation. Group 2 contained 35 cases (23F, 12M) exposed in infancy with a 12-year latent period, and Group 3 contained 26 cases (15F, 11M) exposed at a mean age of 7.6 years with a 6-year latent period before operation (Table 1

**Table 1:** Ages and latent period in years for the patients in the three groups

	**Age at exposure**	**Age at operation**	**Latent period**
Group 1	1.2 (1.0, 1.4)	7.2 (6.8, 7.5)	6.0 (5.7, 6.3)
Group 2	1.2 (1.0, 1.3)	13.5 (13.2, 13.8)	12.3 (12.0, 12.6)
Group 3	7.6 (7.2, 8.0)	13.3 (12.8, 13.8)	5.7 (5.4, 6.0)

). It can be seen that groups one and two have the same age at exposure, that the latent period of group two is twice as long as that for group one, and that groups 2 and 3 have an almost identical mean age at operation. There is no significant difference in the proportion of male and female subjects between the groups. Each group contained a mixture of cases from the different countries.

### Tumour architecture; extent of differentiation

**Figure 3:**
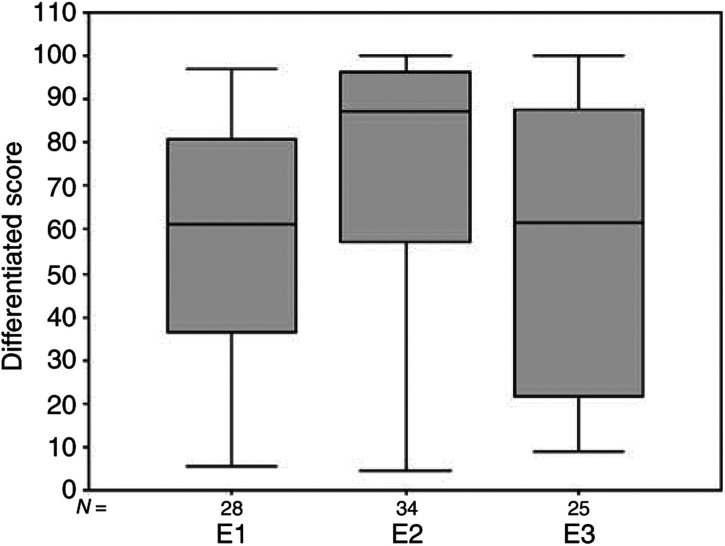
Degree of tumour differentiation (papillary and follicular combined), expressed as % of tumour area, showing the close similarity between the two groups with a short latent period (E1 and E3), and the considerable increase in the % of differentiated tumour in group E2 with a longer latent period. In this and subsequent figures, the box represents the interquartile range. The whiskers are lines that extend from the box to the highest and lowest values, excluding outliers. A line across the box indicates the median. Circles represent outliers (cases between 1.5 and 3 box lengths from the upper or lower end of the box). Stars represent extremes (cases that are beyond 3 box lengths from the upper or lower end of the box).

The extent of differentiation was assessed by combining the more differentiated components (papillary and follicular) together, and comparing these with the less structurally differentiated components (solid and trabecular) combined. The trabecular component was very small (less than 2.3%) and not consistently separable from the solid component. The results show that groups 1 and 3, with a similar latent period, have an almost identical level of structural differentiation (59.1, 95% CI (48.32, 69.79) and 58.0, 95% CI (44.24, 71.71), respectively), while group 2 with a longer latent period shows a greater degree of differentiation (76.1, 95% CI (67.13, 85.05)) (Figure 3

). There is an overall significant difference between the three groups (*χ*^2^=7.494, df=2, *P*=0.024) and for groups 1 *vs* 2 (*z*=−2.709, *P*=0.007), the difference between groups 2 and 3 is of borderline significance (*z*=−1.857, *P*=0.063).

### Tumour architecture, type of differentiation

**Figure 4:**
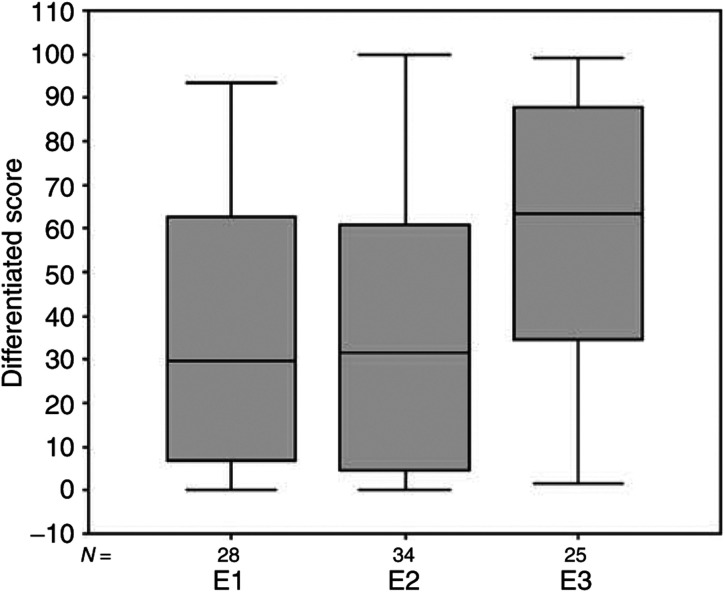
Type of differentiation, expressed as % of differentiated tumour, showing the close similarity between the two groups aged under 2 years at exposure, and the considerable increase in the % of the papillary component in the tumours of group E3, with a mean age of 7.6 years at exposure.

The findings for the direction of differentiation are expressed as the proportion that the papillary component forms of the sum of the two differentiated components, follicular and papillary. It can be seen (Figure 4

) that the findings for groups one and two, both exposed at a young age, but one operated 6 years later and the other 12 years later, are extremely similar. In both, the dominant type of differentiation was follicle formation; the papillary component (36.7, 95% CI (24.44, 48.92) and 36.4, 95% CI (25.17, 47.54), respectively) was minor. In contrast, in group three, exposed at age 6–9 years and showing a short latent period, the dominant type of differentiation was papillary (57.1, 95% CI (43.15, 70.94)). There is an overall significant difference between the groups (*χ*^2^=6.628, df=2, *P*=0.036) and between groups 1 and 3 (*z*=−2.174, *P*=0.030) and groups 2 and 3 (*z*=−2.332, *P*=0.020).

### Extent of invasion

**Figure 5:**
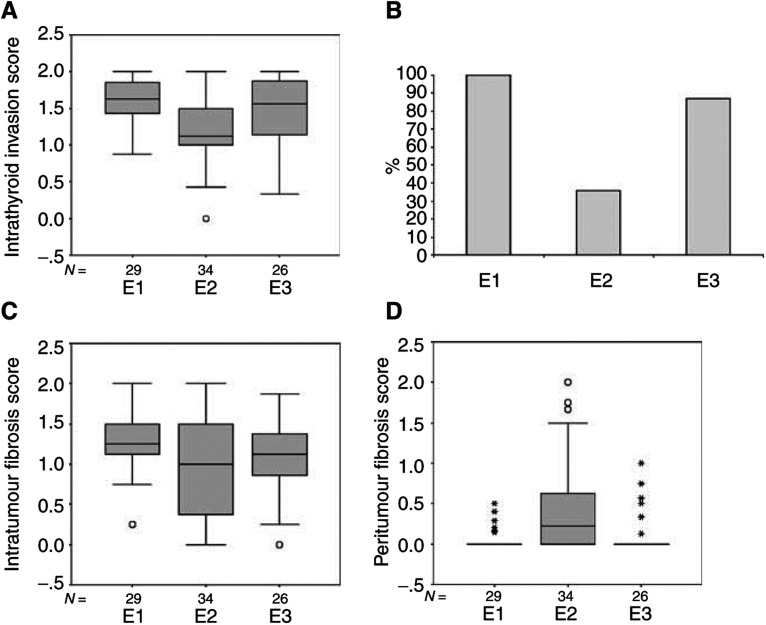
Intra- and extrathyroid invasion (**A**, **B**), and intra- and peritumour fibrosis (**C**, **D**) showing the generally closer relationship of the groups with a shorter latent period (E1 and E3). These groups show more intra- and extrathyroid invasion, and less peritumour fibrosis than the tumours of group E2 with a longer latent period.

Both intra- and extrathyroid invasion were more prominent in the short latency groups (1 and 3) than in group 2. For intrathyroid invasion (Figure 5A

), the mean score was, group one 1.63, 95% CI (1.52, 1.74), group two 1.15, 95% CI (098, 1.32)] and group three 1.48 95% CI (1.30, 1.66)]. Both groups 1 and 3 were significantly higher than group 2 (*z*=−4.091, *P*<0.0001, and *z*=−2.644, *P*=0.008). Extrathyroid invasion was present in 50% of cases in group 1, 26% in group 2 and 50% in group 3. When assessment was restricted to cases where the material included thyroid capsule, invasion was present in 100% of cases in group one, 36% in group two and 87% in group three (Figure 5B). Both short latency groups (1 and 3) showed significantly more invasion than group 2 (1 *vs* 2, *P*<0.001, 3 *vs* 2, *P*<0.001).

### Extent of fibrotic response

Fibrosis within the tumour was assessed separately from peritumour (capsular) fibrosis. For both observations, the two short latent period groups (1 and 3) were similar, differing from the tumours with a longer latent period (group 2). Intratumour fibrosis (Figure 5C) was less marked in group 2 (1.0, 95% CI (0.81, 1.24)) than groups 1 and 3 (1.3, 95% CI (1.15, 1.43) and 1.1, 95% CI (0.87, 1.30)), but the differences were of borderline significance. Peritumour fibrosis (Figure 5D) was significantly higher in group 2 (1.4, 95% CI (1.24, 1.64)) than in groups 1 and 3 (1.1, 95% CI (1.01, 1.11), 1.1, 95% CI (1.01, 1.24)) (1 *vs* 2 *U*=251, *P*=0.0002, 2 *vs* 3 *U*=249.5, *P*=0.002).

## DISCUSSION

Differentiation in the thyroid during development involves the formation of lumina surrounded by polarised cells arising from the solid mass of unpolarised cells present in the thyroglossal anlage. Well-differentiated thyroid carcinomas retain this polarisation around lumina, but the pattern of differentiated growth can be either follicular or papillary. The differentiated thyroid carcinomas derived from the follicular cell are divided into two types on the basis of morphology and biologic behaviour, now known to correlate with the molecular biological findings. The Follicular Carcinomas are usually composed only of follicles, but the Papillary Carcinomas, which form the great majority of the thyroid carcinomas due to the Chernobyl accident, can show solid or follicular as well as papillary patterns of growth, often occurring together in the one tumour. While the solid areas do not show structural differentiation in the form of papillary or follicular architecture, they do show some functional differentiation, with positivity for thyroglobulin protein and mRNA by immunocytochemistry and *in situ* hybridisation. We refer to the solid and trabecular components as structurally less differentiated, but would emphasise that these are part of a papillary carcinoma spectrum and are completely different from the undifferentiated (anaplastic) carcinomas of the thyroid, which lack any trace of structural or functional thyroid differentiation. This pattern also differs from that seen in those tumours referred to as poorly differentiated (e.g. Insular carcinoma), which although capable of producing thyroglobulin behave in a clinically aggressive manner.

In the three groups of post-Chernobyl thyroid Papillary Carcinomas, chosen to vary in their age at exposure, age at operation and latent period, there are highly significant differences in the mean proportions of the solid and differentiated growth patterns. The extent of the solid component is not related to the age at exposure nor to the age at operation, but is closely related to the latent period. This could result if all tumours were growing at approximately the same rate, and were differentiating with time. However if this were the case, tumours with a long latent period would be larger, in fact they are if anything smaller. We suggest that the findings reflect differing tumour growth rates, with those with the most rapid growth rate showing a less mature phenotype and reaching a size that is clinically detectable and leads to operation at an earlier age than tumours with a less rapid growth rate and a more differentiated phenotype. The observations on the extent of invasion and the development of peritumour fibrosis support this; the short latency tumours show a higher level of both intra- and extrathyroid invasion than the long latency group, while the long latency group shows a significantly greater development of peritumour fibrosis, usually associated with less-aggressive tumours. In addition, clinical studies showed that tumours with a long latent period were less aggressive (Farahati *et al*, 2000), although screening could have been relevant, and the study was not controlled for age at exposure. Unfortunately, it was not possible to carry out direct analysis of cells in cycle or of the molecular biology in all of the tumours in our study. However, we have shown in an unpublished study that tumours with a solid morphology have a significantly higher proportion of cells in cycle than tumours with a more structurally differentiated phenotype (Verykoglou *et al*, 2004). We and others have also shown previously that tumours with a more solid phenotype show a higher proportion of cases with a RET-PTC 3 arrangement than do more differentiated tumours (Nikiforov *et al*, 1997; Thomas *et al*, 1999; Rabes *et al*, 2000). In addition, cases that occurred early in this Chernobyl-related thyroid cancer endemic showed a higher proportion of RET-PTC 3-positive tumours; this proportion fell and the proportion of RET-PTC 1-positive cases rose with an increasing latent period (Rabes *et al*, 2000). RET-PTC 3 transgenic mice give rise to thyroid tumours with a more solid phenotype than those seen in RET PTC1 transgenic mice, and *in vitro* RET-PTC3-transfected cells show a higher growth rate than RET-PTC 1-transfected cells (Santoro *et al*, 1996; Powell *et al*, 1998; Basolo *et al*, 2002).

All these observations combine to support the hypothesis that we are seeing successive waves of thyroid tumours occurring after the Chernobyl accident, with the short latent period tumours resulting from oncogenes giving a high growth rate, less structural differentiation and a greater aggressiveness, compared to the later cases with a longer latent period, a lower growth rate, greater differentiation and less aggressiveness; the latter shown clinically and morphologically by less invasion and more capsule formation. It is likely that RET-PTC 3 is one oncogene involved in the short latent period group, with RET-PTC 1 involved in the longer latent period tumours. Tumours lacking any detectable RET rearrangement were rare in the earlier Chernobyl-related tumours studied (Nikiforov *et al*, 1997), in later studies they form nearly half of all cases (Thomas *et al*, 1999).

Some of the early observations were interpreted as suggesting that radiation-related thyroid carcinomas were more aggressive and more often RET-PTC 3 positive than sporadic tumours. It now seems likely that these changes are characteristic of short latency, not of radiation. These conclusions carry the important implication that future cases may be progressively more slowly growing with a progressively more differentiated phenotype. Supporting this is the observation that the papillary thyroid cancers occurring with a long latent period in the population exposed to the atomic bombs in Japan were very well differentiated and showed significantly more peritumour fibrosis than age and sex-matched unexposed cases (Preston and Williams, 2003). In spontaneous thyroid carcinogenesis however, undifferentiated (anaplastic) carcinomas appear to arise from pre-existing differentiated carcinomas, and this could theoretically occur through further somatic mutation in some unresected Chernobyl-related thyroid carcinomas. Continuing study of the Chernobyl-related tumours should allow continuing correlation of molecular biological changes with tumour growth rates, morphology and aggressiveness and may enable treatment to be individualised in a way that is not currently possible. It will also show whether there is an increase in different tumour types which may have a longer latent period than many papillary carcinomas, for example follicular and medullary carcinomas. The creation of a tumour bank, collecting Chernobyl-related thyroid carcinomas and making extracted nucleic acids available for research studies, facilitates such work (Thomas and Williams, 2000).

While the degree of structural differentiation was clearly linked to tumour latency, the type of differentiation was linked to the age at exposure. Many papillary carcinomas contained areas with papillary, and areas with follicular differentiation, one type often dominated. In both groups one and two, exposed at less than 2 years of age, follicular architecture formed over 60% of the differentiated component, while in group three, exposed at a mean age of 7.6 years it formed just over 40%. These differences, between groups three and one, which share similar latent periods, and between groups three and two, which share similar ages at operation, are significant. The reason for this link to age at exposure is not clear. The growth rate of the normal thyroid declines with age, and it is possible that there are age-related changes in the ability of some of the relevant oncogenes either to lead to tumours or to affect tumour growth rate. For example, if there were age-related changes in expression of one of the genes to which the RET tyrosine kinase domain can be rearranged, this could affect the likelihood of that particular rearrangement leading to a clinically detectable tumour, possibly through affecting the chance of acquiring the additional mutations needed to progress to a clinically detectable tumour in an age-related fashion. The sensitivity to radiation-induced thyroid carcinoma is much greater in those who were youngest at exposure, whether following external or internal radiation (Ron *et al*, 1995; Williams, 1996; Cardis *et al*, 1999), and it has been proposed that this is related to the progressive reduction in the number of postexposure cell divisions with increasing age (Williams, 1999). It seems likely that there are complex interactions involving thyroid growth rate, age-related levels of gene expression, type of RET rearrangement, interaction with other oncogenes and possibly other factors that combine to determine sensitivity to thyroid carcinogenesis, and latent period, aggressiveness and pattern of growth of the resulting tumours.

The succession of short latent period aggressive tumours, moderate latent period less-aggressive tumours, and long latent period minimally aggressive tumours suggested by the observations on the Chernobyl and Atom Bomb-related papillary carcinomas is not easily compatible with the proposal that radiation induces genomic instability that continues to generate potentially carcinogenic mutations, including rearrangements, for many cell generations. It does explain the observation that sporadic papillary thyroid carcinomas in young children commonly show a solid morphology and tend to be more aggressive than papillary carcinomas in older children (Harach and Williams, 1995): obviously carcinomas in very young children must necessarily have a short latent period.

The present study has clearly demonstrated that, in these radiation-induced papillary carcinomas, tumour latent period is correlated with tumour morphology and aggressiveness, with short latent period tumours showing considerably more solid less structurally differentiated areas, and higher levels of invasion compared to longer latent period tumours, which show higher levels of more differentiated components, either papillary or follicular, and lower levels of invasion. Surprisingly, the direction of that differentiation, either to a papillary or a follicular pattern of growth, was not correlated with latency or age at operation but with age at exposure. Continuing study of this ongoing markedly increased incidence of thyroid carcinoma due to exposure to fallout from the accident at the Chernobyl nuclear power station in April 1986 should greatly increase our understanding of the biology and natural history of thyroid carcinoma and contribute to knowledge of the carcinogenic process generally.

## References

[bib1] Basolo F, Giannini R, Monaco C, Melillo RM, Carlomagno F, Pancrazi M, Salvatore G, Chiappetta G, Pacini F, Elisei R, Miccoli P, Pinchera A, Fusco A, Santoro M (2002) Potent mitogenicity of the RET-PTC3 oncogene correlates with its presence in tall-cell variant of papillary thyroid carcinoma. Am J Pathol 160: 247–254 11786418 10.1016/S0002-9440(10)64368-4 PMC1867131

[bib2] Baverstock K, Egloff B, Pinchera A, Williams D (1992) Thyroid cancer after Chernobyl. Nature 359: 21–22 10.1038/359021b0 1522880

[bib3] Bogdanova T, Bragarnik M, Tronko ND, Harach HR, Thomas GA, Williams ED (1995) Thyroid cancer in the Ukraine post Chernobyl. In Proceedings of 11th International Thyroid Congress, Toronto

[bib4] Cardis E, Amoros E, Kesminiene A, Malakhova IV, Poliakov SM, Piliptsevitch NN, Demidchik EP, Astakhova LN, Ivanov VK, Konogorov AP, Parshkov EM, Tsyb AF (1999) Observed and predicted thyroid cancer incidence following the Chernobyl accident. In Radiation and Thyroid Cancer, Thomas G, Karaoglou A, Williams ED (eds) pp 395–405. Singapore: World Scientific

[bib5] Farahati J, Demidchik EP, Biko J, Reiners C (2000) Inverse association between age at the time of radiation exposure and extent of disease in cases of radiation-induced childhood thyroid carcinoma in Belarus. Cancer 88: 1470–1475 10717632 10.1002/(sici)1097-0142(20000315)88:6<1470::aid-cncr27>3.0.co;2-w

[bib6] Furmanchuk AW, Averkin JI, Egloff B, Ruchti C, Abelin T, Schappi W, Korotkevich EA (1992) Pathomorphological findings in thyroid cancers of children from the Republic of Belarus. Histopathology 21: 401–408 1452122 10.1111/j.1365-2559.1992.tb00423.x

[bib7] Harach HR, Williams ED (1995) Childhood thyroid cancer in England and Wales. Br J Cancer 72: 777–783 7669594 10.1038/bjc.1995.410 PMC2033913

[bib8] Kazakov VS, Demidchik EP, Astakhova LN (1992) Thyroid cancer after Chernobyl. Nature 359: 21 10.1038/359021a0 1522879

[bib9] Kimura ET, Nikiforova MN, Zhu Z, Knauf JA, Nikiforov YE, Fagin JA (2003) High prevalence of BRAF mutations in thyroid cancer: genetic evidence for constitutive activation of the RET/PTC-RAS-BRAF signaling pathway in papillary thyroid carcinoma. Cancer Res 63: 1454–1457 12670889

[bib10] Nikiforov Y, Gnepp DR (1994) Pediatric thyroid cancer after the Chernobyl disaster. Cancer 74: 748–766 8033057 10.1002/1097-0142(19940715)74:2<748::aid-cncr2820740231>3.0.co;2-h

[bib11] Nikiforov YE, Rowland JM, Bore KE, Montfort-Mungo H, Fagin JA (1997) Distinct pattern of RET oncogene rearrangements in morphological variants of radiation induced and sporadic thyroid papillary carcinomas in children. Cancer Res 57: 1690–1694 9135009

[bib12] Powell DJ, Russell J, Nibu K, Li G, Rhee E, Liao M, Goldstein M, Keane WM, Santoro M, Fusco A, Rothstein JL (1998) The RET-PTC3 oncogene: metastatic solid type papillary carcinomas in murine thyroids. Cancer Res 58: 5523–5528 9850089

[bib13] Preston D, Williams ED (2003) Unpublished data

[bib14] Rabes HM, Demidchik EP, Siderow JD, Lengfelder E, Beimfohr C, Hoelzel D, Klugbauer S (2000) Pattern of radiation induced RET and NTRK1 rearrangements in 191 post Chernobyl papillary carcinomas: biologic, phenotypic and clinical implications. Clin Cancer Res 6: 1093–1103 10741739

[bib15] Ron E, Lubin JH, Shore RE, Mabuchi K, Modan B, Pottern LM, Schneider AB, Tucker MA, Boice JD (1995) Thyroid cancer after exposure to external radiation, a pooled analysis of 7 studies. Radiat Res 141: 259–277 7871153

[bib16] Santoro M, Carlomagno F, Hay ID, Herrmann MA, Grieco M, Melillo R, Pierotti MA, Bongarzone I, Della Porta G, Berger N (1992) RET oncogene activation in human thyroid neoplasms is restricted to the papillary carcinoma subtype. J Clin Invest 89: 1517–1522 1569189 10.1172/JCI115743 PMC443023

[bib17] Santoro M, Chiappetta G, Cerrato A, Salvatore D, Zhang L, Manzo G, Picone A, Portella G, Santelli G, Vecchio G, Fusco A (1996) Development of thyroid papillary carcinomas secondary to tissue specific expression of the RET PTC1 oncogene in transgenic mice. Oncogene 12: 1821–1826 8622903

[bib18] Shigematsu I (1991) The international Chernobyl project technical report. IAEA, Vienna

[bib19] Soares P, Trovisco V, Rocha AS, Lima J, Castro P, Preto A, Maximo V, Botelho T, Seruca R, Sobrinho-Simoes M (2003) BRAF mutations and RET/PTC rearrangements are alternative events in the etiopathogenesis of PTC. Oncogene 22: 4578–4580 12881714 10.1038/sj.onc.1206706

[bib20] Thomas GA, Bunnell H, Cook HA, Williams ED, Nerovnya A, Cherstvoy ED, Tronko ND, Bogdanova TI, Chiappetta G, Viglietto G, Pentimalli F, Salvatore G, Fusco A, Santoro M, Vecchio G (1999) High prevalence of RET PTC rearrangements in Ukrainian and Belarussian post Chernobyl thyroid papillary carcinomas: a strong correlation between RET-PTC 3 and the solid follicular variant. J Clin Endocr Metab 84: 4232–4238 10566678 10.1210/jcem.84.11.6129

[bib21] Thomas GA, Williams ED (2000) Thyroid tumour banks. Science 289: 2283 11041794 10.1126/science.289.5488.2283a

[bib22] Tronko MD, Bogdanova TI, Komissarenko IV, Epstein OV, Oliynyk V, Kovalenko A, Likhtarev IA, Kairo I, Peters SB, LiVolsi VA (1999) Thyroid carcinoma in children and adolescents in Ukraine after the Chernobyl nuclear accident: statistical data and clinicomorphologic characteristics. Cancer 86: 149–156 10391575 10.1002/(sici)1097-0142(19990701)86:1<149::aid-cncr21>3.0.co;2-a

[bib23] UNSCEAR Report (2000) Vol 2, Annex J. United Nations, New York and Geneva

[bib24] Verykoglou M, Thomas GA, Williams ED, Williams GH (2004) Cell cycle proliferation markers in thyroid neoplasia. Unpublished observations

[bib25] Williams ED (1996) Effects on the thyroid in populations exposed to radiation as a result of the Chernobyl accident. In One Decade after Chernobyl, pp 207–230. Vienna: International Atomic Energy Authority

[bib26] Williams ED (1999) Biological mechanisms underlying radiation induction of thyroid carcinoma. In Radiation and Thyroid Cancer, Thomas G, Karaoglou A, Williams ED (eds) pp 177–188. Singapore: World Scientific

[bib27] Williams ED (2002) Cancer after nuclear fallout: lessons from the Chernobyl accident. Nat Rev Cancer 2: 543–549 12094241 10.1038/nrc845

